# Economic Evaluation of Blood Pressure Monitoring Techniques in Patients With Hypertension: A Systematic Review

**DOI:** 10.1001/jamanetworkopen.2023.44372

**Published:** 2023-11-21

**Authors:** Michelle A. Hayek, Theodoros Giannouchos, Mark Lawley, Hye-Chung Kum

**Affiliations:** 1 Population Informatics Lab, Department of Industrial and Systems Engineering, Texas A&M University, College Station; 2 Department of Health Policy and Organization, School of Public Health, The University of Alabama at Birmingham; 3 Department of Industrial and Systems Engineering, Texas A&M University, College Station; 4 Population Informatics Lab, Department of Health Policy and Management, Texas A&M University School of Public Health, College Station

## Abstract

**Importance:**

Blood pressure monitoring is critical to the timely diagnosis and treatment of hypertension. At-home self-monitoring techniques are highly effective in managing high blood pressure; however, evidence regarding the cost-effectiveness of at-home self-monitoring compared with traditional monitoring in clinical settings remains unclear.

**Objective:**

To identify and synthesize published research examining the cost-effectiveness of at-home blood pressure self-monitoring relative to monitoring in a clinical setting among patients with hypertension.

**Evidence Review:**

A systematic literature search of 5 databases (PubMed, MEDLINE, Embase, EconLit, and CINAHL) followed by a backward citation search was conducted in September 2022. Full-text, peer-reviewed articles in English including patients with high blood pressure (systolic blood pressure ≥130 mm Hg and diastolic blood pressure ≥80 mm Hg) at baseline were included. Data from studies comparing at-home self-monitoring with clinical-setting monitoring alternatives were extracted, and the outcomes of interest included incremental cost-effectiveness and cost-utility ratios. Non–peer-reviewed studies or studies with pregnant women and children were excluded. To ensure accuracy and reliability, 2 authors independently evaluated all articles for eligibility and extracted relevant data from the selected articles.

**Findings:**

Of 1607 articles identified from 5 databases, 16 studies met the inclusion criteria. Most studies were conducted in the US (6 [40%]) and in the UK (6 [40%]), and almost all studies (14 [90%]) used a health care insurance system perspective to determine costs. Nearly half the studies used quality-adjusted life-years gained and cost per 1–mm Hg reduction in blood pressure as outcomes. Overall, at-home blood pressure monitoring (HBPM) was found to be more cost-effective than monitoring in a clinical setting, particularly over a minimum 10-year time horizon. Among studies comparing HBPM alone vs 24-hour ambulatory blood pressure monitoring (ABPM) or HBPM combined with additional support or team-based care, the latter were found to be more cost-effective.

**Conclusions and Relevance:**

In this systematic review, at-home blood pressure self-monitoring, particularly using automatic 24-hour continuous blood pressure measurements or combined with additional support or team-based care, demonstrated the potential to be cost-effective long-term compared with care in the physical clinical setting and could thus be prioritized for patients with hypertension from a cost-effectiveness standpoint.

## Key Points

**Question:**

What is the most cost-effective method for measuring blood pressure among patients with hypertension: at-home self-monitoring or in-clinic monitoring?

**Findings:**

In this systematic literature review of 16 studies, at-home self-monitoring was the most cost-effective strategy long-term compared with traditional blood pressure monitoring in clinics, with 24-hour ambulatory blood pressure monitoring and at-home blood pressure monitoring combined with additional support or team-based care being more cost-effective compared with at-home blood pressure monitoring alone.

**Meaning:**

These findings suggest that clinicians, hospitals, health care systems, third-party payers, and other stakeholders should prioritize at-home self-monitoring of blood pressure as the main strategy for blood pressure measurement among patients with hypertension.

## Teaser

This systematic review examines evidence for the cost-effectiveness of at-home blood pressure (BP) self-monitoring vs monitoring in a clinical setting among patients with hypertension.

## Introduction

Hypertension is a significant risk factor for heart attack and stroke and a major cause of premature death.^1^ Globally, nearly 1.3 billion adults have hypertension, with estimated health care expenditures reaching up to $1 trillion over a 10-year period.^2^ However, only approximately 20% of patients with hypertension have their blood pressure (BP) under control.^1^

While BP measurements have traditionally taken place in clinics under the supervision of health care professionals, there has been increasing interest in transitioning to more convenient methods at home that mitigate the burden placed on patients who require regular visits. Self-monitoring at home has emerged as an effective and more convenient approach in monitoring BP levels in comparison with blood pressure measurements taken at a clinic.^3^ Beyond its convenience, this approach informs patients and clinicians about blood pressure changes throughout the day in real time and enables tailored treatment formulation and needed adjustments to mitigate adverse outcomes associated with high BP.^4,5^ However, self-monitoring at home, particularly when combined with additional support and resources (ie, telehealth services, counseling, or collaboration across health care professionals) can also result in added costs to patients, clinicians, hospitals, health care systems, and insurers given the required training and the time spent by clinicians and patients to frequently monitor BP and adjust treatment plans.^6,7^

Multiple studies have explored the costs, benefits, and effects of at-home self-monitoring BP techniques compared with traditional BP measurement in clinical settings.^4,8^ However, to our knowledge, there is no systematic review of the available literature documenting the cost-effectiveness of at-home self-monitoring BP techniques for patients with hypertension over the past decade. In this study, we aimed to systematically gather research findings in the existing literature and to identify whether self-monitoring blood pressure at home is more cost-effective than measuring blood pressure at the clinic. By presenting evidence on the comparative effectiveness of self-monitoring programs, clinicians, stakeholders, and patients can make more informed decisions about the added value of transitioning to self-monitoring BP programs.

## Methods

### Search Strategy

We followed the Preferred Reporting Items for Systematic Reviews and Meta-analyses (PRISMA) and reporting guidelines for economic evaluations to conduct this study.^9,10,11,12^ Five databases were searched (PubMed, CINAHL, MEDLINE, EconLite, and Embase) in September 2022 (eTable 1 in Supplement 1). We further augmented the search using snowballing of references among selected papers.

### Study Selection

Inclusion criteria were established a priori as full-text, peer-reviewed articles in English language including patients with high BP (systolic BP ≥130 mm Hg; diastolic BP ≥80 mm Hg) at baseline without any restrictions on comorbidities. To ensure consistency in the study population, we excluded studies with pregnant women and children. All studies needed to include a comparative economic analysis between at-home BP self-monitoring (intervention) and traditional BP measurements in physical clinical settings (usual care). The intervention included patients self-monitoring their BP at home and transmitting measurements to a health care professional via any means (eg, electronically, short message service, or mail). At-home self-monitoring could involve either periodically taking readings, such as twice each morning and evening during the first week of every month^13^ or 3 times per week^14^ (referred to as at-home BP monitoring [HBPM]) or utilizing 24-hour continuous BP monitoring (referred to as ambulatory BP monitoring [ABPM]), which uses a portable device to automatically measure a patient’s BP at regular intervals, such as every 20 to 30 minutes over a continuous period.^15^

The self-monitoring intervention could also be combined with additional behavioral interventions, such as nurse-tailored behavioral interventions^3,16^ or additional pharmacist care.^17,18,19^ No restrictions were placed on the type of clinician, health care setting, or additional interventions studied. The outcome measures included incremental cost-effectiveness ratios (ICERs) and incremental cost-utility ratios (ICURs).^13,20^ These were obtained from studies conducting cost-effectiveness analysis (CEA) or cost-utility analysis (CUA). CEA is a method of comparing costs with respect to a health outcome measured in nonmonetary values, such as costs added per 1–mm Hg reduction in SBP.^21^ CUA is a method often used interchangeably with CEA and measures the health outcomes using quality-adjusted life-years (QALYs).

### Data Analysis

Two researchers (M.A.H. and T.G.) independently conducted a quality assessment using the Joanna Briggs Institute Critical Appraisal Checklist for Economic Evaluations^22^ to determine the inclusion of each study (eTable 2 in Supplement 1). To ensure accuracy, once the included studies were finalized, data extraction was similarly conducted independently by two researchers using recommended guidelines.^9^ The extracted data included various factors such as participants, study designs, interventions, diagnosis, methods, outcomes, costs, and cost-effectiveness estimates. Data analysis was conducted in Excel version 2309 (Microsoft Corp).

## Results

Initially, a total of 1607 articles were identified from the 5 databases, and after excluding 86 duplicates, 1521 articles were screened based on their title and abstract (eFigure in Supplement 1). Among these, 1483 articles were excluded for not meeting the inclusion criteria, resulting in 38 articles for full-text assessment. During full-text review, 17 articles were excluded. Ten of those (59%) had no economic analysis, 4 (24%) included the intervention as self-monitoring but not in a home-based-setting, and 3 (18%) were excluded based on quality assessment criteria (eTable 2 in Supplement 1). To supplement the search, we then reviewed the references of the 21 identified final articles. This led to the identification of 31 other articles, of which 28 did not meet the title and abstract inclusion criteria. Based on full text review, 3 articles identified from references were added to the original set, resulting in a total of 24 articles^6,13,14,15,16,17,18,19,20,21,23,24,25,26,27,28,29,30,31,32,33,34,35,36^ representing 16 distinct studies (eFigure in Supplement 1).

### Study Quality Assessment Criteria

All of the included studies had a well-defined question and a comprehensive description of alternatives.^6,13,14,15,16,20,21,23,24,25,26,27,28,29,30,31^ Overall, 14 (88%) reported all relevant costs and outcomes for each alternative identified,^6,13,14,15,16,20,21,23,24,25,26,27,28,30^ and all studies established clinical effectiveness.^6,13,14,15,16,20,21,23,24,25,26,27,28,29,30,31^ Most studies (13 [81%]) adequately described costs and outcomes, acknowledged limitations, and addressed concerns regarding the accuracy of the measurements.^6,13,14,15,16,20,21,23,24,26,27,28,30^ Similarly, 12 studies (75%) provided sufficient explanations about how costs and outcomes were measured.^6,13,15,16,20,21,23,24,26,27,28,30^ Differential timing was taken into account in all of the studies.^6,13,14,15,16,20,21,23,24,25,26,27,28,29,30,31^ An incremental analysis of costs and outcomes was conducted in 15 studies (94%).^6,13,14,15,20,21,23,24,25,26,27,28,29,30,31^ A sensitivity analysis was conducted in 15 studies (94%).^6,13,14,15,16,20,21,23,24,25,26,27,28,29,30^ The majority of the studies provided a comprehensive assessment of the cost-effectiveness of the intervention over the control.^6,13,14,15,16,20,21,23,24,25,26,27,28,29,30^ All of the studies adequately described the study setting and discussed the issue of generalizing the results to a different setting with similar characteristics.^6,13,14,15,16,20,21,23,24,25,26,27,28,29,30,31^

### Main Study Characteristics

Of 16 studies, 7 were trials,^6,14,16,20,21,25,29^ 1 was a quasi-experimental study,^31^ and 8 were simulation models, of which 7 were Markov models^13,15,23,24,26,28,30^ and 1 was a microsimulation model.^27^ All interventions were compared with usual care (UC). The definition of usual care had minor differences but was generally considered as patients monitored per usual clinical care from their clinician without self–BP monitoring.^31^ All articles included an intervention in which patients self-measured their BP at home using an electronic device and transferred their BP results to clinicians at a different location (ie, HBPM). HBPM was classified into 3 categories^37^: HBPM alone, HBPM with additional support (eg, basic website, online patient portals, or telephone consultations with clinicians or nurses^17^), and HBPM within team-based care (TBC), which involved more labor-intensive efforts, often combined with other behavioral interventions, such as pharmacist care^17,18,19^ or nurse behavioral interventions.^3,16^ Blood pressure measured over a continuous 24-hour period was referred to as ABPM^15,26,27,28^ (Table 1).^38,39^ Detailed characteristics of each article can be found in Table 2. Seven articles reported the comparison of more than 1 intervention,^13,15,16,18,26,27,28^ of which 4 included ABPM^15,26,27,28^ (Table 3).

**Table 1.** Different BP Measurement Methods

Methods/acronym	Description
HBPM alone	Patients self-measure BP at home without additional support or interventions
HBPM with additional support	Patients self-measure BP at home with basic additional resources or support
HBPM within TBC	Patients self-measure BP at home within a TBC approach, involving more intensive efforts and often combined with other behavioral interventions
ABPM	Patients’ blood pressure measured continuously at home over a 24-h period
UC	Traditional patient BP measurements at the clinical setting

Abbreviations: ABPM, ambulatory blood pressure monitoring; BP, blood pressure; HBPM, home blood pressure monitoring; TBC, team-based care; UC, usual care.

**Table 2.** General Characteristics of the Identified Articles

Source	Country	Eligibility diagnosis	Method	Time horizon	CEA or CUA	Perspective	Year of unit costs	Direct medical costs/direct program costs^a^
McManus et al,^35^ 2018; Monahan et al,^13^ 2019	UK	Hypertension	Markov model	65 y	CUA	NHS and Personal Social Services	2015-2016	Yes/yes
Bosworth et al,^36^ 2008; Reed et al,^16^ 2010	US	Hypertension	2 × 2 Factorial design	24 mo	CEA	Societal	2008	Yes/yes
Green et al,^18^ 2008; Fishman et al,^20^ 2013	US	Hypertension	3-Group RCT	12 mo	CEA	Health care	2009	No/yes
Margolis et al,^19^ 2013; Dehmer et al,^14^ 2018	US	Hypertension	2-Group cluster RCT	18 mo	CEA	Health care	2008-2009	Yes/no
McManus et al,^33^ 2010; Kaambwa et al,^23^ 2014	UK	Hypertension	Markov model	35 y	CUA	Health care	2009-2010	Yes/yes
Ionov et al,^24^ 2021	Russia	Hypertension	Markov model	10 y	Both	Ministry of Health of the Russian Federation	2018	Yes/yes
Madsen et al,^34^ 2008; Madsen et al,^6^ 2011	Denmark	Hypertension	Unblinded RCT	6 mo	CEA	Health care	2007	Yes/yes
McKinstry et al,^21^ 2013	UK	Hypertension	Pragmatic, prospective, parallel-group RCT with blinded outcome assessments	6 mo	CEA	NHS	2009-2010	Yes/yes
Magid et al,^17^ 2013; Billups et al,^25^ 2014	US	Hypertension	Pragmatic RCT	6 mo	CEA	Health care	2009	Yes/yes
Lovibond et al,^26^ 2011	UK	Hypertension	Markov model	60 y	CUA	NHS and Personal Social Service	2009-2010	Yes/yes
Green et al,^27^ 2022	US	Hypertension	Microsimulation model	Until patients reach the age of 89 y or death	CUA	Health care	2021	Yes/yes
Beyhaghi and Viera,^28^ 2019	US	Hypertension	Markov model	Until patients reach the age of 85 y	CUA	Health care	2017	Yes/no
Shah et al,^15^ 2023	Australia	Hypertension	Markov model	Lifetime	Both	Australian government	2019-2020	Yes/yes
McManus et al,^29^ 2005	UK	Hypertension	Unblinded RCT	12 mo	CEA	Health care	2001-2002	Yes/yes
McManus et al,^32^ 2014; Penaloza-Ramos et al,^30^ 2016	UK	Hypertension with diabetes or CKD and/or previous cardiovascular disease	Markov model	30 y	CUA	NHS and Personal Social Service	2011-2012	Yes/yes
Teo et al,^31^ 2021	Singapore	Hypertension or hypertension with hyperlipidemia	Open-label, quasi-experimental study	6 mo	Both	Societal	2018	Yes/no

Abbreviations: CEA, cost-effectiveness analysis; CKD, chronic kidney disease; CUA, cost-utility analysis; NHS, National Health Service; RCT, randomized clinical trial.

^a^ Direct medical costs were defined as medical-related health costs, such as cost of acute or chronic cardiovascular disease events^30^; medication costs^24^; or inpatient, outpatient, or emergency department visit costs.^25^ Direct program costs were defined as costs related to the intervention including training, equipment, and office units.^20^

**Table 3.** Analysis Methods and Outcome Measures in Cost Analysis Studies

Source	Control	Intervention(s)	Outcome measure	WTP threshold	Sensitivity analysis	ICER per intervention(s) per outcome measure	Probability of CE per WTP
McManus et al,^35^ 2018; Monahan et al,^13^ 2019	UC	HBPM alone; HBPM with additional support	Cost per QALY	£20 000/QALY	Yes	HBPM alone vs UC: £3035; HBPM with additional support vs HBPM alone: £17 424	HBPM alone: 38%; HBPM with additional support: 51%
Bosworth et al,^36^ 2008; Reed et al,^16^ 2010	UC	HBPM; TBC; HBPM within TBC	Cost per 1–mm Hg BP reduction; cost per life-year gained	NR	Yes	HBPM with TBC vs UC: cost per 1–mm Hg SBP reduction, $107 in direct medical costs and $297 when including patient time costs; cost per 1–mm Hg DBP reduction, NR; cost per life-year gained: $4169	NR
Green et al,^18^ 2008; Fishman et al,^20^ 2013	UC	HBPM with additional support; HBPM within TBC	Cost per person achieving BP control; cost per 1–mm Hg BP reduction; cost per change in life expectancy achieved through greater control of hypertension	NR	Yes	HBPM with additional support vs UC: cost per person achieving BP control, no significant difference; cost per 1–mm Hg SBP reduction, $23.8 (95% CI, $21.3 to $26.2); cost per 1–mm Hg DBP reduction, no significant difference; cost per change in life expectancy, no significant difference; HBPM within TBC vs HBPM with additional support: cost per person achieving BP control, $16.7 (95% CI, $15.4 to $17.9); cost per 1–mm Hg SBP reduction, $65.3 (95% CI, $59.9 tp $70.7); cost per 1–mm Hg DBP reduction, $114.8 (95% CI, $111.9 to $117.7); cost per change in life expectancy among males, $1850 (95% CI, $1635.7 to $2064.2); among females, $2220 (95% CI, $1745.1 to $2694.9)	NR
Margolis et al,^19^ 2013; Dehmer et al,^14^ 2018	UC	HBPM within TBC	Cost per person achieving BP control; cost per 1–mm Hg BP reduction	NR	Yes	Cost per person achieving BP control, $7337; cost per 1–mm Hg SBP reduction, $139; cost per 1–mm Hg DBP reduction, $265	NR
McManus et al,^33^ 2010; Kaambwa et al,^23^ 2014	UC	HBPM with additional support	Cost per QALY	£20 000 to £30 000/QALY	Yes	Men, £1,624; women, £4923	99% of iterations for men and women
Ionov et al,^24^ 2021	UC	HBPM with additional support	Cost per 1–mm Hg BP reduction; cost per QALY	300 000 RUB/1–mm Hg decrease	Yes	Cost per 1–mm Hg BP reduction, 731.1 RUB; cost per QALY, 275 179 RUB	76%
Madsen et al,^34^ 2008; Madsen et al,^6^ 2011	UC	HBPM with additional support	Cost per 1–mm Hg BP reduction	NR	Yes	Cost per 1–mm Hg reduction in SBP, 256 DKK (95% CI, −860 to 4544 DKK); in DBP, 655 DKK (95% CI, −674 to 69 315 DKK)	NR
McKinstry et al,^21^ 2013	UC	HBPM with additional support	Cost per 1–mm Hg BP reduction	NR	Yes	£25.6 (95% CI, £16.1 to £46.7)	NR
Magid et al,^17^ 2013; Billups et al,^25^ 2014	UC	HBPM within TBC	Cost per 1–mm Hg BP reduction; cost per person achieving BP control; cost per life-year gained	NR	Yes	Cost per 1–mm Hg BP reduction, $20.5; cost per person achieving BP control; $1331; cost per life-year gained, $3330	NR
Lovibond et al,^26^ 2011	UC	HBPM alone; ABPM	Cost per QALY	£20 000 to £30 000/QALY	Yes	ABPM dominates UC and HBPM alone	ABPM in almost all iterations
Green et al,^27^ 2022	UC	UC and HBPM alone; UC and ABPM	Cost per QALY	$50 000 to $150 000/QALY	Yes	UC and HBPM alone vs UC, UC dominates; UC and ABPM vs UC, $85 164	At WTP of $50 000, UC dominant; at WTP of $100 000, ABPM: 66%; at WTP of $150 000, ABPM: 89.5%
Beyhaghi and Viera,^28^ 2019	UC	HBPM alone; ABPM	Cost per QAD	$50 000/QALY	Yes	HBPM alone vs UC: UC dominates; ABPM vs HBPM alone: ABPM dominates	ABPM in almost all iterations in men and women <80 y of age
Shah et al,^15^ 2023	UC	HBPM alone; ABPM	Cost per QALY; cost per life-year gained	Cost per QALY, A$50 000; cost per life-year gained, A$28 003	Yes	HBPM alone vs UC, HBPM alone dominates both outcomes; ABPM vs HBPM alone, ABPM dominates both outcomes	ABPM in almost all iterations
McManus et al,^29^ 2005	UC	HBPM alone and self-measured BP at GP	Cost per 1–mm Hg BP reduction	NR	Yes	£5.1 (95% CI, −£7.2 to £19.1)	NR
McManus et al,^32^ 2014; Penaloza-Ramos et al,^30^ 2016	UC	HBPM with additional support	Cost per QALY	£20 000 to £30 000/QALY	Yes	Dominates	99%
Teo et al,^31^ 2021	UC	HBPM within TBC	Cost per person achieving BP control; cost per QALY	<1 GDP per capita per QALY; GDP assumed S$78 000 or S$58 000	No	Cost per person achieving BP control, S$468.6; cost per QALY, S$23 935.1	NR

Abbreviations: ABPM, ambulatory blood pressure monitoring; BP, blood pressure; CE, cost-effective; DBP, diastolic blood pressure; GP, general practitioner; HBPM, home blood pressure monitoring; NR, not reported; SBP, systolic blood pressure; QAD, quality-adjusted day; QALY, quality-adjusted life-year; TBC, team-based care; UC, usual care; WTP, willingness-to-pay threshold.

### Economic Analysis

Among the 16 studies, 7 utilized CEA,^6,14,16,20,21,25,29^ 6 used CUA,^13,23,26,27,28,30^ and 3 utilized both CUA and CEA.^15,24,31^ A societal perspective was only reported in 2 studies,^16,31^ and the rest were conducted using a health care system perspective.^6,13,14,15,20,21,23,24,25,26,27,28,29,30^ The types of costs reported in each article were different, with 3 studies reporting direct medical costs only,^14,28,31^ 1 reporting only program costs,^20^ and 12 reporting both^6,13,15,16,21,23,24,25,26,27,29,30^ (Table 2).

### Cost-Effectiveness Outcomes

#### Outcome Measure

The health outcome measures differed across studies and included cost per QALY in 8 studies,^13,15,23,24,26,27,30,31^ cost per life-year saved in 3 studies,^15,16,25^ cost per 1–mm Hg reduction in BP in 7 studies,^6,14,20,21,24,25,29^ cost per improved BP control in 3 studies,^14,20,25^ and cost per quality-adjusted days (QADs) in 1 study.^28^ In 9 studies (4 of which were conducted in the UK,^13,23,26,30^ 2 in the US,^27,28^ and the rest in Russia,^24^ Australia,^15^ and Singapore^31^), a willingness to pay threshold (WTP) to determine whether an intervention was cost-effective was mentioned. A sensitivity analysis was conducted in 15 studies (94%)^6,13,14,15,16,20,21,23,24,25,26,27,28,29,30^ (Table 3).

#### Cost-Effective Interventions

Of the 16 identified studies, 9 reported that an at-home BP monitoring intervention (HBPM or ABPM) had an ICER within the WTP thresholds, indicating the intervention as cost-effective compared with clinical UC.^13,15,23,24,26,27,28,30,31^ Of the remaining 7, all were studies with time periods ranging from 6 to 24 months.^6,14,16,20,21,25,29^ Of the 12 identified studies that compared the cost-effectiveness of HBPM vs UC, 5 (42%) reported the intervention as being cost-effective.^13,23,24,30,31^ Notably, all 5 of these HBPM interventions included either additional support^13,23,24,30^ or TBC^31^ alongside HBPM alone. In the single study that compared HBPM alone vs HBPM with additional support vs UC, HBPM with additional support was the most cost-effective option, with the reported ICER of HBPM alone vs usual care being £3035 and the reported ICER of HBPM alone vs HBPM with additional support being £17 424 at a WTP threshold of £20 000 per QALY gained.^13^

The remaining 4 studies compared HBPM alone vs ABPM vs UC, and all 4 reported ABPM as being the most cost-effective intervention over both HBPM alone and usual care.^15,26,27,28^ Of 8 simulation models using a time period of 10 years or more, all 8 reported that either HBPM or ABPM were more cost-effective than UC^13,15,23,24,26,27,28,30^ (Table 3).

## Discussion

This systematic review examined the cost-effectiveness of HBPM in comparison with UC. Among the identified studies, approximately 60% determined at-home self-monitoring (HBPM or ABPM) to be cost-effective over usual care, particularly long term. HBPM was also found to be cost-effective when combined with additional support or within TBC compared with HBPM alone. For studies comparing HBPM alone with UC and with ABPM, ABPM was found to be the most cost-effective method.

The clinical effectiveness of at-home self-monitoring is well-documented in most of the retrieved studies, even among those that used trial time periods of 6 to 24 months and showed improvements in either BP control or reductions in mean BP levels.^17,18,19,21,24,29,31,32,33,34,40^ However, our findings suggest that at-home BP self-monitoring interventions, particularly ABPM and HBPM with additional support or TBC, exhibit superior incremental gains in the long run compared with UC but may not demonstrate well-justified costs given short-term differential outcomes over periods of 6 to 24 months. This is not surprising given the high upfront fixed costs (ie, equipment, training) to implement an at-home self-monitoring intervention and the clinical course and time required to achieve substantially improved health outcomes and to avoid adverse events related to uncontrolled BP. Combined, our findings suggest that clinicians, hospitals, health care systems, third-party payers, and other stakeholders should consider the long-term incremental benefits and improvements in patients’ BP, quality of life, and reductions in adverse outcomes, which might well justify both the upfront and longer-term costs of more resource-intensive at-home self-monitoring methods over traditional usual care and at-home self-monitoring only. The documented cost-effectiveness of BP self-monitoring at-home with additional support or TBC aligns with previous studies that conducted reviews and synthesized costs and benefits of self-monitoring methods across various diseases and settings.^4,37^

At-home self-monitoring, particularly ABPM, might further enable the detection of masked and white-coat hypertension. These terms refer to the detection of elevated BP in a clinical setting (known as white-coat hypertension) and low BP using HBPM or ABPM, or vice versa (known as masked hypertension).^41^ It is currently estimated that 17.1 million adults in the United States have masked hypertension.^27^ This is crucial because one-time or limited office measurements, despite being the traditional method, may vary considerably due to different techniques and methods used, particularly in the presence of a physician or nurse.^42^ Hence, at-home self-monitoring might be more accurate and allow tailored interventions early on, which are critical to control BP and mitigate the associated risks of masked hypertension or prevent unnecessary treatment uptake due to white-coat hypertension. At-home self-monitoring also has the potential to improve patient and clinician satisfaction, particularly in terms of the convenience of recording and sharing BP measurements from home vs going to the clinic. Moreover, self-monitoring at home facilitates easier communication with the health care professional when needed, particularly if coupled with additional behavioral interventions or additional support and resources, which allow patients to receive timely advice during consultations and enable the health care team to gain a better understanding of the patient’s condition.^31^ This can thereby increase patient motivation to monitor BP, which would consequently improve the care provided by enabling the health care professionals to better tailor their treatment approach and ultimately enhance patient outcomes.^43^

Although this study was based on a systematic literature search and the findings are reported and synthesized in a robust manner, we note that the detailed comparison of many study results and their generalizability across different perspectives and settings remains challenging, particularly due to high variability and inconsistency in reported costs. Almost all the studies included in our review did not account for indirect and intangible costs, such as productivity losses or patient out-of-pocket costs. In addition, given the limited number of studies found in this systematic review that used a societal perspective, future work is needed to conduct cost-effectiveness analyses and evaluations across the various types of BP measurement methods from the societal perspective and incorporate indirect and intangible patient-level costs. Including such costs might significantly affect the incremental cost differences across competing alternatives and thus findings and future recommendations. Along these lines, we did not find any studies comparing ABPM with HBPM with additional support or TBC—all 3 of which were found to be more cost-effective than UC and HBPM alone in separate analyses. Future studies might consider conducting cost-effectiveness analyses with such comparisons. Finally, we note that patient experiences and preferences related to the differences between various home-based care and monitoring systems vs traditional clinical based care can critically affect patients’ adherence and, in turn, prevention, and timely treatment initiation and should thus also be considered when determining the WTP pay for such interventions.

### Strengths and Limitations

This study has several strengths, including a thorough systematic review, data collection, dual independent review of the processes at all stages, utilizing recommended guidelines for conducting the systematic review,^9,10,11,22^ and the representation of the results in tabular summaries. In addition, this is the most up-to-date study that systematically gathers and synthesizes the available literature on the cost-effectiveness of at-home self-monitoring for patients with hypertension, to our knowledge.

Nevertheless, this study also has some limitations. First, most studies included a single stakeholder perspective and thus might not be generalizable. Second, the reported costs across the studies varied due to the types of cost data being included. The details of types of costs included were not consistently provided, making it challenging to compare findings among different studies. In addition, most studies utilized different definitions of cost inputs, with medical and program costs defined differently. More research is needed to further evaluate the cost-effectiveness of self-monitoring BP at home in comparison with usual care using longer time periods with costs reported in consistent and clear manner and across multiple perspectives.

## Conclusions

In this systematic review, at-home blood pressure monitoring demonstrated the potential to be cost-effective over the long-term in comparison with the more usual clinical patient care. Compared with HBPM alone, ABPM and HBPM with additional support or TBC were also found to be more cost-effective. Future work is needed to compare these alternatives directly from a cost-effectiveness standpoint and to provide clinicians, stakeholders, and patients with more evidence to prioritize specific home-based BP programs.

## Notes

Supplement 1. **eTable 1.** Search Strategy **eFigure.** Search Strategy Results **eTable 2.** Additional Information on Article Selection and Quality Assessment
eReferences.

Supplement 2.
Data Sharing Statement
