# Supplementary material for: Economic Evaluation of Blood Pressure Monitoring Techniques in Patients With Hypertension: A Systematic Review
Source: JAMA Netw Open. 2023 Nov 21;6(11):e2344372. doi: 10.1001/jamanetworkopen.2023.44372 (PMC10663963; doi:10.1001/jamanetworkopen.2023.44372)
Supplement: Supplement 1. — eTable 1. Search Strategy eFigure. Search Strategy Results eTable 2. Additional Information on Article Selection and Quality Assessment eReferences. [file jamanetwopen-e2344372-s001.pdf]

Supplemental Online Content

Hayek MA, Giannouchos T, Lawley M, Kum H. Economic evaluation of blood pressure monitoring techniques in patients with hypertension: a systematic review. *JAMA Netw Open.* 2023;6(11):e2344372. doi:10.1001/jamanetworkopen.2023.44372

eTable 1. Search Strategy

eFigure. Search Strategy Results

eTable 2. Additional Information on Article Selection and Quality Assessment

eReferences.

This supplemental material has been provided by the authors to give readers additional information about their work.

© 2023 Hayek MA et al. *JAMA Network Open.*

eTable 1. Search Strategy

Query	Limiters/Expanders
("self-management" OR telemonitoring OR "tele- monitoring" OR "remote monitoring" OR "self- monitoring" OR "self monitoring" OR "remote sens*") AND ("Cost-Benefit Analysis" OR "Costs and Cost Analysis" OR economic*)	Limiters - Full Text; Scholarly (Peer Reviewed) Journals Expanders - Apply equivalent subjects Narrow by Language: -English Search modes - Boolean/Phrase

eFigure. Search Strategy Results

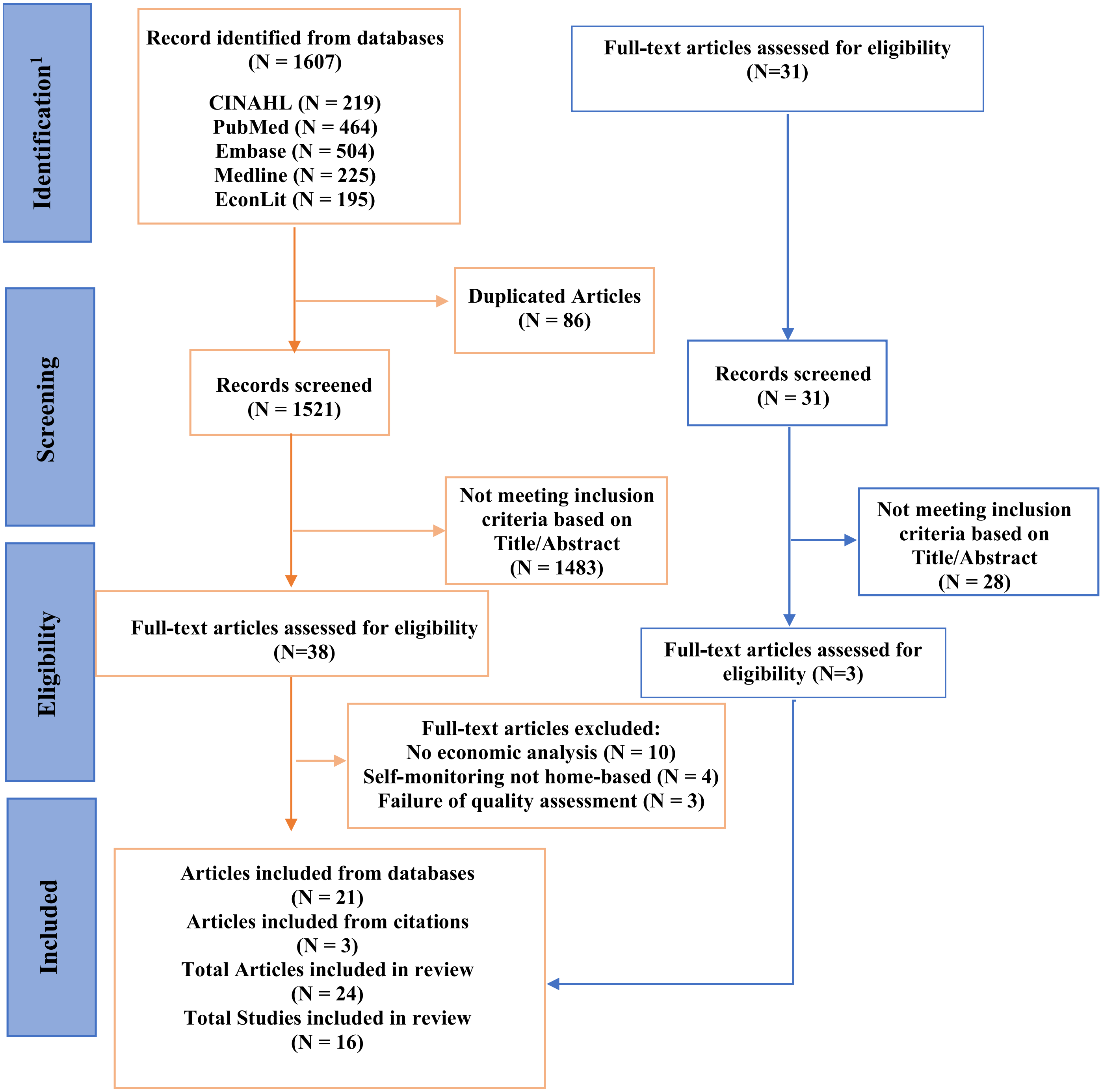

© 2023 Hayek MA et al. *JAMA Network Open.*

eTable 2. Additional Information on Article Selection and Quality Assessment

Author, Year	Q1*	Q2	Q3	Q4	Q5	Q6	Q7	Q8	Q9	Q10	Q11	Overall Appraisal
McManus et al, [2] 2018; Monahan et al, [3] 2019	Yes	Yes	Yes	Yes	Yes	Yes	Yes	Yes	Yes	Yes	Yes	Include
Bosworth et al, [4] 2008; Reed et al, [5] 2010	Yes	Yes	Yes	Yes	Yes	Yes	Yes	Unclear	Yes	Yes	Yes	Include
Fishman et al, [6] 2013; Green et al, [7] 2008	Yes	Yes	Yes	Yes	Yes	Yes	Yes	Yes	Yes	Yes	Yes	Include
Dehmer et al, [8] 2018; Margolis et al, [9] 2013	Yes	Yes	Yes	Yes	Yes	Unclear	Yes	Yes	Yes	Yes	Yes	Include
Kaambwa et al, [10] 2014; McManus et al, [11] 2010	Yes	Yes	Yes	Yes	Yes	Yes	Yes	Yes	Yes	Yes	Yes	Include
Friedman et al,[12] 1996	Yes	Yes	No	Yes	Unclear	Unclear	Unclear	Yes	Yes	Yes	Yes	Exclude
Ionov et al, [13] 2021	Yes	Yes	Yes	Yes	Yes	Yes	Yes	Yes	Yes	Yes	Yes	Include
Madsen et al,[14] 2008; Maden et al,[15] 2011	Yes	Yes	Yes	Yes	Yes	Yes	Yes	Yes	Yes	Yes	Yes	Include
McKinstry et al, [16] 2013	Yes	Yes	Yes	Yes	Yes	Yes	Yes	Yes	Yes	Yes	Yes	Include
Billups et al, [17] 2014; Magid et al, [18] 2013	Yes	Yes	Yes	Yes	Unclear	Unclear	Yes	Yes	Yes	Yes	Yes	Include

© 2023 Hayek MA et al. *JAMA Network Open.*

eTable 2. Additional Information on Article Selection and Quality Assessment (continued)

Lovibond et al, [19] 2011	Yes	Yes	Yes	Yes	Yes	Yes	Yes	Yes	Yes	Yes	Yes	Include
Arrieta et al,[20] 2014	Yes	No	No	Yes	Yes	Yes	Yes	No	Yes	No	Unclear	Exclude
Green et al, [21] 2022	Yes	Yes	Yes	Yes	Yes	Yes	Yes	Yes	Yes	Yes	Yes	Include
Beyhaghi; Viera, [22] 2019	Yes	Yes	Yes	Yes	Yes	Yes	Yes	Yes	Yes	Yes	Yes	Include
Shah et al, [23] 2023	Yes	Yes	Yes	Yes	Yes	Yes	Yes	Yes	Yes	Yes	Yes	Include
McManus et al, [24] 2005	Yes	Yes	Unclear	Yes	Unclear	No	Yes	Yes	Yes	Yes	Yes	Include
McManus et al, [25] 2021	Yes	Yes	Yes	Yes	Unclear	Unclear	Yes	Yes	Yes	Unclear	Unclear	Exclude
McManus et al,[26] 2014; Penaloza-Ramos et al, [27] 2016	Yes	Yes	Yes	Yes	Yes	Yes	Yes	Yes	Yes	Yes	Yes	Include
Teo et al, [28] 2021	Yes	Yes	No	Yes	Unclear	Unclear	Yes	Yes	No	Unclear	Yes	Include

Abbreviations: *Q1 – Q11: Question 1 – Question 11 from the JBI Critical Appraisal Checklist for Economic Evaluations[29]

Question 1: Is there a well-defined question?
Question 2: Is there a comprehensive description of alternatives?
Question 3: Are all important and relevant costs and outcomes for each alternative identified?
Question 4: Has clinical effectiveness been established?
Question 5: Are costs and outcomes measured accurately?
Question 6: Are costs and outcomes valued credibly?
Question 7: Are costs and outcomes adjusted for differential timing?
Question 8: Is there an incremental analysis of costs and consequences?
Question 9: Were sensitivity analyses conducted to investigate uncertainty in estimates of cost or consequences?
Question 10: Do study results include all issues of concern to users?
Question 11: Are the results generalizable to the setting of interest in the review?

© 2023 Hayek MA et al. *JAMA Network Open.*

eReferences

1. Page MJ, McKenzie JE, Bossuyt PM, et al. The PRISMA 2020 statement: An updated guideline for reporting systematic reviews. PLoS Med. 2021;18(3). doi:10.1371/JOURNAL.PMED.1003583
2. McManus RJ, Mant J, Franssen M, et al. Efficacy of self-monitored blood pressure, with or without telemonitoring, for titration of antihypertensive medication (TASMINH4): an unmasked randomised controlled trial. The Lancet. 2018;391(10124):949-959. doi:10.1016/S0140-6736(18)30309-X
3. Monahan M, Jowett S, Nickless A, et al. Cost-Effectiveness of Telemonitoring and Self-Monitoring of Blood Pressure for Antihypertensive Titration in Primary Care (TASMINH4). Hypertension. 2019;73(6):1231-1239. doi:10.1161/HYPERTENSIONAHA.118.12415
4. Bosworth HB, Olsen MK, Neary A, Orr M, Grubber J, Svetkey L, Adams M, Oddone EZ. Take Control of Your Blood Pressure (TCYB) study: a multifactorial tailored behavioral and educational intervention for achieving blood pressure control. Patient Educ Couns. 2008 Mar;70(3):338-47. doi: 10.1016/j.pec.2007.11.014.
5. Reed SD, Li Y, Oddone EZ, et al. Economic evaluation of home blood pressure monitoring with or without telephonic behavioral self-management in patients with hypertension. Am J Hypertens. 2010;23(2):142-148. doi:10.1038/ajh.2009.215
6. Fishman PA, Cook AJ, Anderson ML, et al. Improving BP Control Through Electronic Communications: An Economic Evaluation. Am J Manag Care. 2013;19(9):709. Accessed December 9, 2022. /pmc/articles/PMC3938103/
7. Green BB, Cook AJ, Ralston JD, et al. Effectiveness of Home Blood Pressure Monitoring, Web Communication, and Pharmacist Care on Hypertension Control: A Randomized Controlled Trial. JAMA. 2008;299(24):2857-2867. doi:10.1001/JAMA.299.24.2857
8. Dehmer SP, Maciosek M V., Trower NK, et al. Economic evaluation of the home blood pressure telemonitoring and pharmacist case management to control hypertension (Hyperlink) trial. Journal of the American College of Clinical Pharmacy. 2018;1(1):21-30. doi:10.1002/JAC5.1001
9. Margolis KL, Asche SE, Bergdall AR, et al. Effect of home blood pressure telemonitoring and pharmacist management on blood pressure control a cluster randomized clinical trial. JAMA. 2013;310(1):46-56. doi:10.1001/jama.2013.6549
10. Kaambwa B, Bryan S, Jowett S, et al. Telemonitoring and self-management in the control of hypertension (TASMINH2): A cost-effectiveness analysis. Eur J Prev Cardiol. 2014;21(12):1517-1530. doi:10.1177/2047487313501886
11. McManus RJ, Mant J, Bray EP, et al. Telemonitoring and self-management in the control of hypertension (TASMINH2): A randomised controlled trial. The Lancet. 2010;376(9736):163-172. doi:10.1016/S0140-6736(10)60964-6
12. Friedman RH, Kazis LE, Jette A, et al. A telecommunications system for monitoring and counseling patients with hypertension impact on medication adherence and blood pressure control. Am J Hypertens. 1996;9(4 I):285-292. doi:10.1016/0895-7061(95)00353-3
13. Ionov M V., Zhukova O V., Yudina YS, et al. Value-based approach to blood pressure telemonitoring and remote counseling in hypertensive patients. Blood Press. 2021;30(1):20-30. doi:10.1080/08037051.2020.1813015
14. Madsen LB, Kirkegaard P, Pedersen EB. Blood pressure control during telemonitoring of home blood pressure. A randomized controlled trial during 6 months. Blood Press. 2008;17(2):78-86. doi:10.1080/08037050801915468
15. Madsen LB, Christiansen T, Kirkegaard P, Pedersen EB. Economic evaluation of home blood pressure telemonitoring: A randomized controlled trial. Blood Press. 2011;20(2):117-125. doi:10.3109/08037051.2010.532306
16. McKinstry B, Hanley J, Wild S, et al. Telemonitoring based service redesign for the management of uncontrolled hypertension: multicentre randomised controlled trial. BMJ. 2013;346(7913). doi:10.1136/BMJ.F3030

© 2023 Hayek MA et al. *JAMA Network Open.*

17. Billups SJ, Moore LR, Olson KL, Magid DJ. Cost-effectiveness evaluation of a home blood pressure monitoring program. Am J Manag Care. 2014;20(9):e380-e387.
18. Magid DJ, Olson KL, Billups SJ, Wagner NM, Lyons EE, Kroner BA. A pharmacist-led, American heart association Heart360 web-enabled home blood pressure monitoring program. Circ Cardiovasc Qual Outcomes. 2013;6(2):157-163. doi:10.1161/CIRCOUTCOMES.112.968172/FORMAT/EPUB
19. Lovibond K, Jowett S, Barton P, et al. Cost-effectiveness of options for the diagnosis of high blood pressure in primary care: A modelling study. The Lancet. 2011;378(9798):1219-1230. doi:10.1016/S0140-6736(11)61184-7
20. Arrieta A, Woods JR, Qiao N, Jay SJ. Cost-benefit analysis of home blood pressure monitoring in hypertension diagnosis and treatment: An insurer perspective. Hypertension. 2014;64(4):891-896. doi:10.1161/HYPERTENSIONAHA.114.03780
21. Green MB, Shimbo D, Schwartz JE, et al. Cost-Effectiveness of Masked Hypertension Screening and Treatment in US Adults With Suspected Masked Hypertension: A Simulation Study. Am J Hypertens. 2022;35(8):752-762. doi:10.1093/AJH/HPAC071
22. Beyhaghi H, Viera AJ. Comparative Cost-Effectiveness of Clinic, Home, or Ambulatory Blood Pressure Measurement for Hypertension Diagnosis in US Adults: A Modeling Study. Hypertension. 2019;73(1):121-131. doi:10.1161/HYPERTENSIONAHA.118.11715
23. Shah KK, Willson M, Agresta B, Morton RL. Cost Effectiveness of Ambulatory Blood Pressure Monitoring Compared with Home or Clinic Blood Pressure Monitoring for Diagnosing Hypertension in Australia. Pharmacoecon Open. 2023. doi:10.1007/S41669-022-00364-0
24. McManus RJ, Mant J, Roalfe A, et al. Targets and self monitoring in hypertension: randomised controlled trial and cost effectiveness analysis. BMJ. 2005;331(7515):493-496. doi:10.1136/BMJ.38558.393669.E0
25. McManus RJ, Little P, Stuart B, et al. Home and Online Management and Evaluation of Blood Pressure (HOME BP) using a digital intervention in poorly controlled hypertension: Randomised controlled trial. The BMJ. 2021;372. doi:10.1136/BMJ.M4858
26. McManus RJ, Mant J, Haque MS, et al. Effect of self-monitoring and medication self-titration on systolic blood pressure in hypertensive patients at high risk of cardiovascular disease: the TASMIN-SR randomized clinical trial. JAMA. 2014;312(8):799-808. doi:10.1001/JAMA.2014.10057
27. Penaloza-Ramos MC, Jowett S, Mant J, et al. Cost-effectiveness of self-management of blood pressure in hypertensive patients over 70 years with suboptimal control and established cardiovascular disease or additional cardiovascular risk diseases (TASMIN-SR). Eur J Prev Cardiol. 2016;23(9):902-912. doi:10.1177/2047487315618784
28. Teo VHY, Teo SH, Burkill SM, et al. Effects of technology-enabled blood pressure monitoring in primary care: A quasi-experimental trial. J Telemed Telecare. Published online July 30, 2021. doi:10.1177/1357633X211031780/ASSET/IMAGES/LARGE/10.1177_1357633X211031780-FIG3.JPEG
29. Critical Appraisal Tools | JBI. Accessed March 25, 2023. https://jbi.global/critical-appraisal-tools
30. Bosworth HB, Powers BJ, Olsen MK, et al. Home Blood Pressure Management and Improved Blood Pressure Control: Results From a Randomized Controlled Trial. Arch Intern Med. 2011;171(13):1173-1180. doi:10.1001/ARCHINTERNMED.2011.276
31. De Guzman KR, Snoswell CL, Taylor ML, Gray LC, Caffery LJ. Economic Evaluations of Remote Patient Monitoring for Chronic Disease: A Systematic Review. Value Health. 2022;25(6):897-913. doi:10.1016/J.JVAL.2021.12.001
32. Gaziano TA, Bitton A, Anand S, Weinstein MC. The global cost of nonoptimal blood pressure. J Hypertens. 2009;27(7):1472-1477. doi:10.1097/HJH.0B013E32832A9BA3
33. Husereau D, Drummond M, Petrou S, et al. Consolidated Health Economic Evaluation Reporting Standards (CHEERS) statement. BMJ. 2013;346. doi:10.1136/BMJ.F1049

© 2023 Hayek MA et al. *JAMA Network Open.*

34. Jacob V, Chattopadhyay SK, Proia KK, et al. Economics of Self-Measured Blood Pressure Monitoring: A Community Guide Systematic Review. Am J Prev Med. 2017;53(3):e105-e113. doi:10.1016/J.AMEPRE.2017.03.002
35. Kirkland EB, Heincelman M, Bishu KG, et al. Trends in Healthcare Expenditures Among US Adults With Hypertension: National Estimates, 2003-2014. J Am Heart Assoc. 2018;7(11). doi:10.1161/JAHA.118.008731
36. Monahan M, Jowett S, Lovibond K, et al. Predicting out-of-office blood pressure in the clinic for the diagnosis of hypertension in primary care: An economic evaluation. Hypertension. 2018;71(2):250-261. doi:10.1161/HYPERTENSIONAHA.117.10244
37. Neumann PJ, Sanders GD, Russel LB, Siegel JE, Ganiats TG. Cost-Effectiveness in Health and Medicine. 2nd ed. Oxford University Press; 2016.
38. O'Brien E, Asmar R, Beilin L, et al. European Society of Hypertension recommendations for conventional, ambulatory and home blood pressure measurement. J Hypertens. 2003;21(5):821-848. doi:10.1097/00004872-200305000-00001
39. Omboni S, Gazzola T, Carabelli G, Parati G. Clinical usefulness and cost effectiveness of home blood pressure telemonitoring:Meta-analysis of randomized controlled studies. J Hypertens. 2013;31(3):455-468. doi:10.1097/HJH.0b013e32835ca8dd
40. Owens P, Atkins N, O'Brien E. Diagnosis of White Coat Hypertension by Ambulatory Blood Pressure Monitoring. Hypertension. 1999;34(2):267-272. doi:10.1161/01.HYP.34.2.267
41. Rabi DM, McBrien KA, Sapir-Pichhadze R, et al. Hypertension Canada's 2020 Comprehensive Guidelines for the Prevention, Diagnosis, Risk Assessment, and Treatment of Hypertension in Adults and Children. Can J Cardiol. 2020;36(5):596-624. doi:10.1016/J.CJCA.2020.02.086
42. Sanders GD, Neumann PJ, Basu A, et al. Recommendations for Conduct, Methodological Practices, and Reporting of Cost-effectiveness Analyses: Second Panel on Cost-Effectiveness in Health and Medicine. JAMA. 2016;316(10):1093-1103. doi:10.1001/JAMA.2016.12195
43. Soghikian K, Casper SM, Fireman BH, et al. Home blood pressure monitoring. Effect on use of medical services and medical care costs. Med Care. 1992;30(9):855-865. doi:10.1097/00005650-199209000-00009
44. Uhlig K, Patel K, Ip S, Kitsios GD, Balk EM. Self-measured blood pressure monitoring in the management of hypertension: A systematic review and meta-analysis. Ann Intern Med. 2013;159(3):185-194. doi:10.7326/0003-4819-159-3-201308060-00008/SUPPL_FILE/AIME201308060-00008_SUPPLEMENT2.PDF
45. Whelton PK, Carey RM, Aronow WS, et al. 2017 ACC/AHA/AAPA/ABC/ACPM/AGS/APhA/ASH/ASPC/NMA/PCNA Guideline for the Prevention, Detection, Evaluation, and Management of High Blood Pressure in Adults: A Report of the American College of Cardiology/American Heart Association Task Force on Clinical Practice Guidelines. Hypertension. 2018;71(6):E13-E115. doi:10.1161/HYP.0000000000000065
46. World Health Organization. Accessed May 21, 2023. https://www.who.int/news-room/fact-sheets/detail/hypertension

© 2023 Hayek MA et al. *JAMA Network Open.*
