# Supplementary material for: Economic Evaluation of Blood Pressure Monitoring Techniques in Patients With Hypertension: A Systematic Review
Source: JAMA Netw Open. 2023 Nov 21;6(11):e2344372. doi: 10.1001/jamanetworkopen.2023.44372 (PMC10663963; doi:10.1001/jamanetworkopen.2023.44372)
Supplement: Supplement 2. — Data Sharing Statement [file jamanetwopen-e2344372-s002.pdf]

Data Sharing Statement

Hayek. Economic Evaluation of Blood Pressure Monitoring Techniques in Patients With Hypertension. *JAMA Netw Open*. Published November 21, 2023.
doi:10.1001/jamanetworkopen.2023.44372

Data
Data available: No

Additional Information
Explanation for why data not available: No private data was used. All information is cited using online literature
